# Assessing the Biological Significance of Gene Expression Signatures and Co-Expression Modules by Studying Their Network Properties

**DOI:** 10.1371/journal.pone.0017474

**Published:** 2011-03-07

**Authors:** Pablo Minguez, Joaquin Dopazo

**Affiliations:** 1 Department of Bioinformatics and Genomics, Centro de Investigación Príncipe Felipe (CIPF), Valencia, Spain; 2 CIBER de Enfermedades Raras (CIBERER), Valencia, Spain; 3 Functional Genomics Node, (INB) at CIPF, Valencia, Spain; University of Birmingham, United Kingdom

## Abstract

Microarray experiments have been extensively used to define signatures, which are sets of genes that can be considered markers of experimental conditions (typically diseases). Paradoxically, in spite of the apparent functional role that might be attributed to such gene sets, signatures do not seem to be reproducible across experiments. Given the close relationship between function and protein interaction, network properties can be used to study to what extent signatures are composed of genes whose resulting proteins show a considerable level of interaction (and consequently a putative common functional role).

We have analysed 618 signatures and 507 modules of co-expression in cancer looking for significant values of four main protein-protein interaction (PPI) network parameters: connection degree, cluster coefficient, betweenness and number of components. A total of 3904 gene ontology (GO) modules, 146 KEGG pathways, and 263 Biocarta pathways have been used as functional modules of reference.

Co-expression modules found in microarray experiments display a high level of connectivity, similar to the one shown by conventional modules based on functional definitions (GO, KEGG and Biocarta). A general observation for all the classes studied is that the networks formed by the modules improve their topological parameters when an external protein is allowed to be introduced within the paths (up to the 70% of GO modules show network parameters beyond the random expectation). This fact suggests that functional definitions are incomplete and some genes might still be missing. Conversely, signatures are clearly not capturing the altered functions in the corresponding studies. This is probably because the way in which the genes have been selected in the signatures is too conservative. These results suggest that gene selection methods which take into account relationships among genes should be superior to methods that assume independence among genes outside their functional contexts.

## Introduction

Recently, there exist a growing interest in the definition and use of molecular signatures [1]. These are sets of genes that can be considered markers of diseases, experimental conditions, etc. The changes in the cell functionality provoked by the differential expression of such gene modules must be, to some extent, responsible for the phenotypic differences observed in the experiments. However, the concept of signature has been often criticized. These are currently defined as genes with a significant differential expression between the trait of interest and a control condition. The low sensitivity of the tests for differential expression [2] used to define such signatures produces the well known effect of the instability in its definition [3] and concerns on the reproducibility or results across laboratories or platforms [4].

On the other hand, experimental results from microarrays have brought about the definition of *de facto* co-expression modules [5], [6]. Typically, biclustering techniques are used to define groups of genes that co-express under a certain range of experimental conditions or in a number of samples. Such modules have been demonstrated to be enriched by functionally-related genes and, generally speaking, are thought to be playing some functional role (despite still uncharacterized in some occasions) [7].

It is widely accepted that most of the biological functionality of the cell arises from complex interactions between their molecular components that define operational interacting entities or modules [8]. Understanding the structure and the dynamics of the complex intercellular network of interactions that contribute to the structure and function of a living cell is one of the main challenges in functional genomics [9] and constitutes the objective of systems biology [10]. However, our knowledge of such modules is still very limited and comes from initiatives such as Gene Ontology (GO) [11] or repositories like the Kioto Encyclopedia of Genes and genomes (KEGG) [12] or Biocarta pathways [13]. Such initiatives provide conceptual definitions for functionally-related gene modules, usually supervised by curators and based on different types of evidences.

Intuitively, the notion of module makes reference to a number of cell components (commonly genes or proteins) that collectively accomplish a relatively autonomous and delimited function [8]. It is then expected that genes in a functional module display a certain degree of coordinate expression [14] and that the corresponding gene products are located in physical proximity within the cell, most probably in physical contact in many cases. Actually, it has been reported several times that genes with similar expression profiles are likely to encode interacting proteins [15], [16]. Thus, making use of these concepts protein function has been predicted from gene co-expression [17], [18] and protein-protein interactions [19], [20], [21] data.

In an attempt to evaluate the functional significance of the different modules defined in gene expression experiments (signatures and co-expression modules) we have explored their internal connectivity. Thus, de facto definitions of co-expression modules in cancer as well as signatures of different nature (up- and down-regulated genes from cancer and non-cancer studies), taken from the L2L resource [22] were mapped onto the scaffold of the interactome. Different network parameters were evaluated and tested for the corresponding sub-networks to assess the degree of internal structure in such modules. In order to calibrate whether the degree of connectivity found corresponded to what it was expectable from modules with a real functional role or not, known functional modules (defined as GO, KEGG and Biocarta categories) were used as reference.

The results obtained clearly indicate that signatures obtained from expression profiling experiments contain little network structure. Contrarily, co-expression modules seem to display a higher level of internal network structure similar to the level found in conventional functional modules.

## Results

### PPI network enrichment in reference module definitions: Gene Ontology, KEGG and Biocarta

A list of 8462 sets of transcripts sharing a particular Gene Ontology term was generated by considering. Here, we consider any gene as member of the GO module at which it is annotated as well as all of the parent modules too [23]. Of a total of 8462 GO modules, those with less than three components (4284 GO terms) or more than 200 (274 GO terms) were discarded from the analysis given the difficulties for building empirical random distributions outside of this range. The final analysis was performed over 3904 GO modules. For every GO module two Minimal Connected Networks (MCNs) were computed and tested by PPI network enrichment method [24]: one of them including only proteins annotated with the GO and a second one in which the introduction of one external node in the network is allowed (see methods). Thus, the distributions of values of degree, betweenness and clustering coefficient for all the nodes and the number of components of the network were calculated and compared to their random expectations.

Table 1 shows the percentage of GO terms showing unexpectedly high or low values for these parameters. The distribution of values for the connection degree parameter is significantly above of the random expectations for more than one third of the GO terms. The number of components is significantly lower than expected by chance in one fourth of the GO terms. If the analysis is conducted allowing one extra node, these figures rise significantly (see Table 1), suggesting that some terms could be incomplete in their original definitions.

**Table 1:** Percentage of significant network parameters (p<0.05) in the different conceptual module definitions.

	GO		KEGG		BioCarta	
	module	+node	module	+node	module	+node
**Betweenness**	10.10	36.53	17.90	48.97	11.80	38.80
**Connections degree**	36.30	71.52	44.80	59.31	51.30	66.50
**Cluster coefficient**	16.00	22.21	26.20	31.03	1.90	14.80
**Number of components**	25.40	51.92	30.30	52.41	25.90	40.30

Percentages of lists in every module definition with a significant p-value compared to random distributions for each network parameter obtained for the members of the module (module column) and allowing for an extra node (+node column). The comparisons performed are betweenness, connections degree and clustering coefficient greater than random expectations and number of components lower than random expectations.

When the results are segregated into the GO main categories, biological process, molecular function and cellular component, the last one seem to present more internal network structure (see Table 2). Specifically, in the case of connection degree, half of the cellular component modules present values higher than the random expectation. On the opposite side, the molecular function category presents a low internal network structure.

**Table 2:** Percentage of significant network parameters (p<0.05) in the different GO module definitions.

	GO main categories		
	Biological process	Molecular function	Cellular component
**Betweenness**	10.1	6.2	19.8
**Connections degree**	37.5	28.1	50.1
**Cluster coefficient**	17.7	11.7	17.6
**Number of components**	25.8	20.2	36.0

Percentages of lists in every module definition with a significant p-value compared to random distributions for each network parameter. The comparisons performed are betweenness, connections degree and clustering coefficient greater than random expectations and number of components lower than random expectations.

**Figure 1:**
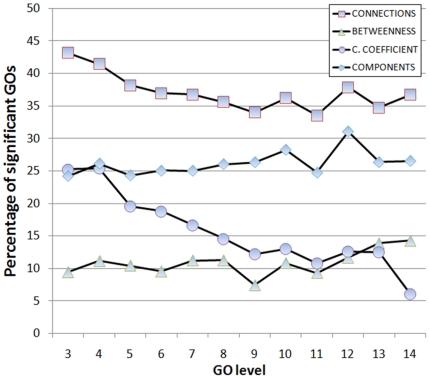
Relationship of different network properties with the GO level.

The specificity of the GO term is represented by the level: the deeper the level the more specific the definition. Network properties do not seem to be especially affected by the GO level, and remain constant over a wide range (approximately from 3 to 14, that covers the range of application of the method) (see Figure 1). Clustering coefficient seems to slightly escape to this trend by reducing its value as the GO depth increases. This is probably an effect of the reduction in the number of proteins as the GO depth increases that affects more to this network property.

It is worth mentioning that GO terms contain different types of conceptualizations of cell functionality. Consequently, some of them do not make direct reference to entities that could be assimilated to a functional module for which one can expect a certain level of co-expression and/or interaction. There is also a certain level of redundancy given by the GO levels that can be observed in the position in the DAG hierarchy that the significant terms have (many are located along branches). Taking these facts into account it can be corroborated that GO terms mainly represent highly interacting gene modules.

The same analysis of network parameters was conducted for human KEGG pathways. Out of a total of 188 KEGG pathways, 41 of them were composed by less than 3 transcripts and 1 by more than 200 transcripts. Thus a total of 146 MCNs were computed. In the case of Biocarta pathways, from a total of 313 pathways there were 50 of them with less than 3 transcripts so, after removing them, 263 MCNs were generated. Table 1 shows the results obtained. KEGG and Biocarta modules display even higher values of connection degree (in the last case up to half of the modules show a value for the parameter significantly higher than the random expectation). The most remarkable difference was found in the cluster coefficient parameter, which was extremely low in the case of Biocarta modules (only 1.9% of the modules show a value higher than the random expectation, which rises to a 14.8% if one extra node is allowed). This might be a consequence of the nature of the modules represented in Biocarta, which mostly contain signalling networks for which a high connection degree but not a high clustering coefficient is expectable. Again, if an extra node is allowed, the values of the network parameters raise significantly (see Table 1). This observation suggests again that some terms could be incomplete in their original definitions. Alternatively, some proteins that do not belong to the modules could be connecting different parts of the module, helping them to be physically close.

### PPI network enrichment in co-expression modules and signatures defined by microarray experiments

A total of 618 signatures (differentially expressed genes) from human microarray experimental results and 507 modules of co-expression in cancer were downloaded from L2L [22]. A PPI network enrichment analysis was conducted for each of the modules. It has previously been described that proteins not selected as part of signatures in microarray experiments were related to disease due to its inclusion into a network of PPIs [25], [26]. Thus, the analyses here were conducted allowing one extra node in the MCN calculations.

The most remarkable observation is that the proportion of co-expression modules with significant network parameters is higher than the equivalent values in the signatures and more similar to the corresponding values observed for GO, KEGG or Biocarta (see Table 3). These results suggest that co-expression modules could be representing functional modules of similar nature than the ones defined by GO, KEGG or, Biocarta.

**Table 3:** Percentage of significant (p<0.05) network parameters in the different module definitions.

	Functional categories			co-expression modules	Signatures			
	GO	KEGG	BioCarta		Cancer	Non-cancer	Up-regulated	Down-regulated
**Betweenness**	36.53	48.97	32.59	34.9	20.44	19.85	21.02	20.00
**Connections degree**	71.52	59.31	55.91	52.10	29.33	31.23	30.57	30.00
**Cluster coefficient**	22.21	31.03	12.46	13.40	7.56	2.91	2.87	2.87
**Number of components**	51.92	52.41	33.87	38.00	18.22	18.40	15.61	15.61

Percentages of lists in every module definition with a significant p-value compared to random distributions for each network parameter. The values were obtained for the members of the module allowing the inclusion of one extra node. The comparisons performed are betweenness, connections degree and clustering coefficient greater than random expectations and number of components lower than random expectations.

On the other hand signatures most probably constitute incomplete descriptions of the functions activated or deactivated in the different scenarios studied. Signatures have been obtained by applying individual, independent tests to any of the genes represented in the microarray followed by a correction for multiple testing. It is known that this results in a considerable lack of statistical power in the testing schema [27]. Obviously, the way in which the relevant genes in the signature are defined is implicitly conditioning the functional interpretation of the whole experiment. Paradoxically, many of the biological properties used to define gene modules (function, regulation, etc.) implies the existence of a high level of cooperative activity among them (in practical terms co-expression [5], [18], [28] and protein interactions), while most of the tests used to select relevant genes assume independence in the behaviours of the genes imposing thus an artificial threshold with a unfavourable effect in the results [27]. This also explains why co-expression modules have more internal network properties.

### Comparison of network properties among the different module definitions

When the network properties (measured as the number of significant different network parameters) are compared across all the module definitions, GO and KEGG display a greater amount of network structure (see Table 3). In fact, KEGG shows higher betweenness and clustering coefficient while GO seems to be more connected although less structured. Both GO and KEGG display the highest proportions of modules (more than 50%) that have significantly less components than it would be expected just by chance, which is again presumable if an underlying network structure exists. Surprisingly Biocarta pathways present fewer cases with significant network parameters than GO or KEGG, being more comparable to what it was observed for co-expression modules. Signatures have been sub-classified according to two criteria: cancer versus non-cancer and up-regulated versus down-regulated. In general, signatures have a low number of cases with significant network parameters when compared to the other module definitions. It is worth noticing that cancer signatures and down-regulated signatures have higher values of clustering coefficient that non-cancer signatures and up-regulated signatures, suggesting the existence of a more interconnected network in the genes differentially expressed in these experimental conditions, which is in agreement with previous observations [29].

**Figure 2:**
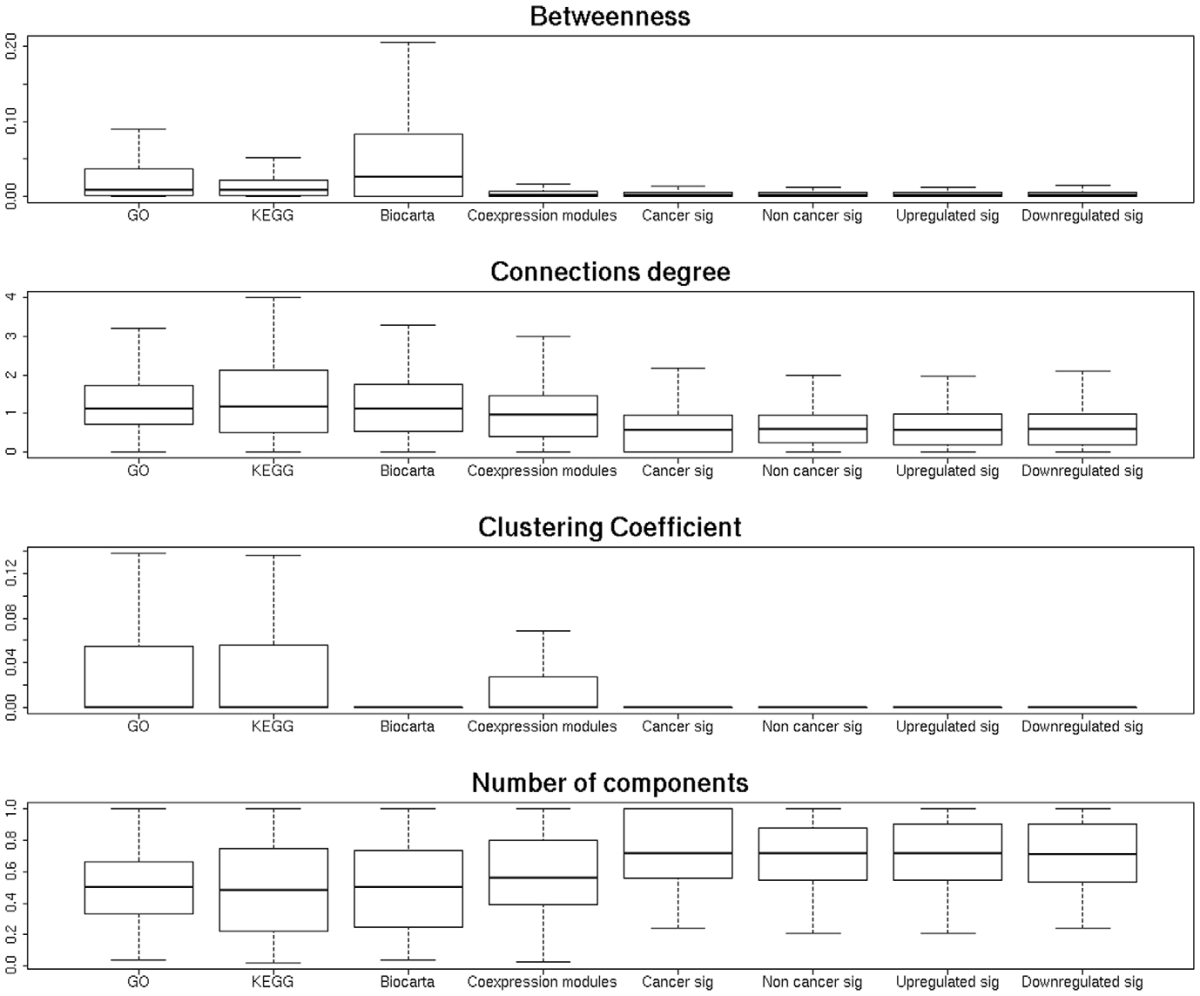
Boxplots representing the distribution of the different network parameters in the different gene module definitions.

When the distributions of the network parameters are studied the results are similar: co-expression modules seem to be in between functional modules of reference (GO, KEGG and Biocarta) and signatures (see Figure 2). Actually, when the distributions of parameter values are compared, the values of betweenness, clustering coefficient and connectivity are significantly higher for the reference modules than for co-expression modules or signatures whereas the number of components is significantly lower, which clearly demonstrates the higher network structure of the former with respect to the later (Table 4). The same pattern of significant comparisons is observed when co-expression modules are compared to signatures, which documents a more compact network structure for co-expression modules (Table 4).

**Table 4:** P-values corresponding to the comparisons of distributions of network parameters across several module definitions by means of a two-tailed Kolmogorov-Smirnov test.

	GO versus Co-expression modules	GO versus signatures	Co-expresion modules versus Signatures
**Betweenness**	2.2×10^−16^	2.2×10^−16^	0.0004724
Connections degree	3.331×10^−16^	2.2×10^−16^	2.2×10^−16^
**Cluster coefficient**	1.763×10^−05^	2.2×10^−16^	2.183×10^−07^
**Number of components**	4.619×10^−14^	2.2×10^−16^	2.2×10^−16^

In all the cases the distribution for the first member was demonstrated to be significantly greater that the one for the second member, except in the case of the parameter “Number of components” in which the first member of the comparison was significantly lower than the second one.

**Figure 3:**
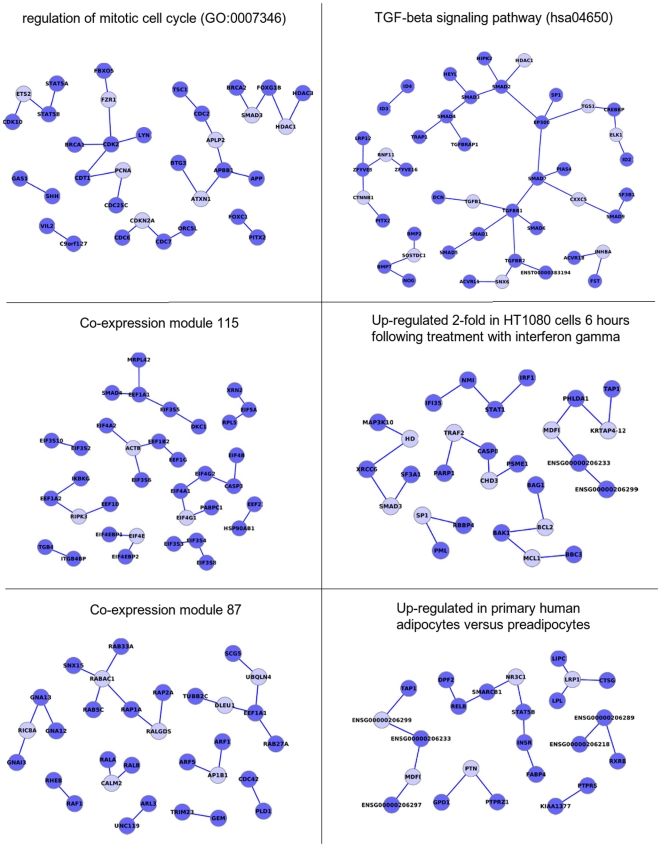
Examples of networks with significant parameters obtained for different module definitions. The networks have been obtained for different types of modules (GO, KEGG, signatures and co-expression modules). All the networks have been chosen with a similar number of nodes (around 50 genes/proteins). In the networks represented, additional nodes connecting nodes in the lists were allowed. Table 5 shows the significance of the network parameters obtained for the modules. Nodes originally in the list are represented in dark blue and extended nodes in pale blue.

Figure 3 shows an example of the PPI networks underlying different modules. The modules represented have been chosen to have about 50 nodes (genes/proteins). Although there are only examples, their network properties are paradigmatic of each type of module. Both, the GO module (regulation of mitotic cell cycle) and the KEGG module (TGF-beta signalling pathway), are highly connected and their connections are wired in a way that the level of betweenness is high. The density of the connections, as represented by the clustering coefficient, is also high in both cases, although superior in the case of the KEGG module. The coexpression modules 115, corresponding to prostate and renal cancers (see http://ai.stanford.edu/~erans/cancer/modules/module_115.html) enriched in genes related to translation activity and protein biosynthesis, and 87, found in hematologic cancers (see http://ai.stanford.edu/~erans/cancer/modules/module_87.html) and enriched in genes of translation activity too, are highly connected and present a high betweenness but in both cases the clustering coefficient is not significantly different from the random expectation. The results obtained for the signatures are unequal. While the signature obtained for genes differentially regulated by gamma interferon [30] has network properties similar to what was observed for the coexpression modules, in the other extreme, the signature obtained for human adipocites [31] does not present any significant network property. Table 5 shows the significance of the network parameters of the PPI networks shown in Figure 3. Files S1 and S2 contain the values of the network parameters for all the signatures and coexpression modules analyzed.

**Table 5:** Significance (p-value) of the network parameters measured for the different network properties in the different modules.

	GO:0007346	Hsa04350	Module 115	ifn_gamma_up	Module 87	adip_human_up
**Betweenness**	<0.0001	<0.0001	<0.0001	0.0001	0.0008	*0.1419*
**Connections**	<0.0001	<0.0001	<0.0001	<0.0001	<0.0001	*0.1205*
**Clustering coefficient**	0.0058	<0.0001	*0.5088*	*0.9959*	*0.9966*	*0.3573*

Non significant values (p>0.05) are in italics.

## Discussion

### What are co-expression modules and signatures composed of?

Co-expression modules and signatures are supposed to explain to some extent the functional differences between the phenotypes or experimental conditions compared. Functional enrichment analysis is often used to confirm the functional roles of such modules. Here we have carried out an extensive analysis of these modules derived from many experiments to know to what extent this relationship module-function is true and what is the predominant nature of the functionality. To achieve so we have studied the enrichment in both GO terms (by a conventional functional enrichment method [23]) and PPI (as described in the methods section) in the 665 signatures and 507 co-expression modules used above.

**Figure 4:**
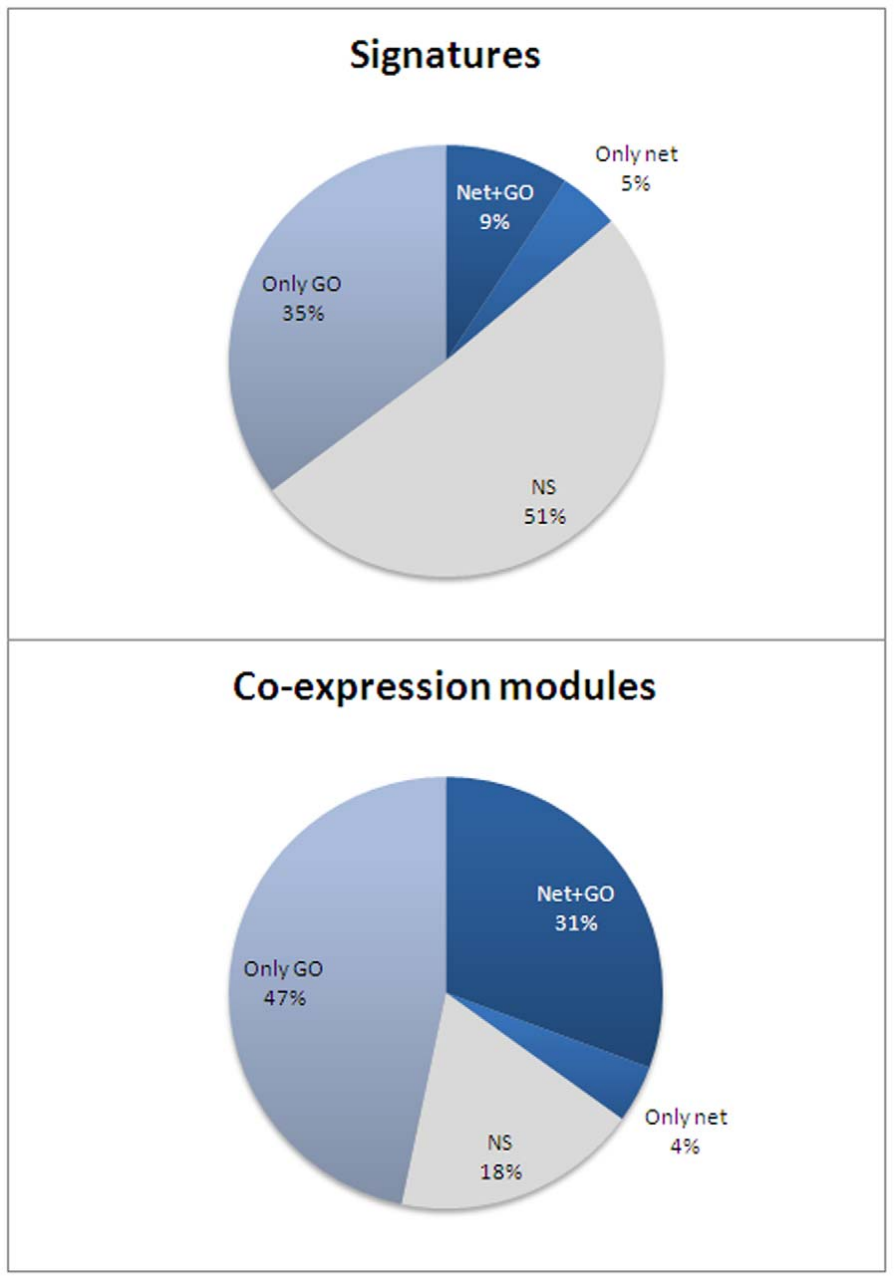
Analysis of over-representation of GO terms and significance in network parameters in signatures and co-expression modules. The analysis was carried out on 665 signatures and 507 co-expression modules. Different sectors in the pie charts represent the percentages of the cases in which only a GO term was found as significantly over-represented (Only GO), cases in which only some network parameter was significant (Only net), cases in which both GO and net parameters were significant (Net+GO) and cases in which nothing was found as significant.

The results are summarized in Figure 4. The first two obvious conclusions are i) co-expression modules are by far more enriched in both functional terms (GO) and network structure than signatures are (82% versus 49%), and ii) both, co-expression modules and signatures are more enriched by functional terms than in network structure. This suggests that co-expression modules are capturing part of the functionality of the cell while signatures fail to do so at the same extent.

Although most of the cases significantly enriched in network parameters were also enriched in GO terms there is still a small amount of them (5% of signatures and 4% of co-expression modules) that are exclusively enriched by network structure. It is also remarkable that in a large number of the cases a functional enrichment has been found with no detectable significant network structure.

### Conclusions

Conventional functional modules (GO, KEGG or Biocarta) can be considered representatives of cell activity components. It is not surprising, thus, that a relatively large amount of network structure can be detected in them through the corresponding network parameters. Despite the fact that functional modules are not perfectly defined and that the description of the human interactome is far from being definitive [32] the results obtained provide a quantitative relationship between network structure and functionality. When co-expression modules are analysed, the results show a moderate degree of network structure (although lower than the degree of structure displayed by the conventional functional modules). Finding a certain level of structure, despite moderate, is in agreement with the well known relationship between co-expression and function [14]. The co-expressing gene products of the modules are expected to be located in physical proximity within the cells, most probably in physical contact in many cases [15], [16]. Actually protein function has been inferred from gene co-expression [17], [18] and protein-protein interactions [19], [20], [21] data.

When signatures are analysed following the same scheme, the number of those with significant values for network parameters is unexpectedly low. The reasons for this observation are unclear but the low sensitivity of the tests for differential expression [2] that produces the well known effect of the instability in the signatures [3] and the questionable reproducibility or results [4] must probably be among the causes.

It has recently been reported that only about 30% of the modules defined by GO terms and 57% of the modules defined by KEGG pathways display an internal correlation higher than the expected by chance [14]. These proportions fit well with the relative proportions of GO and KEGG with a connection degree higher than the random expectation (Table 1). This fact, in combination with the study of enrichment in functional modules (GO, KEGG and Biocarta) suggests that co-expression modules are capturing the functionality of the cell.

However, signatures seem to provide only an incomplete representation of the functionality of the cell. This is most probably a consequence of the testing strategy used for defining them, which is too conservative [33]. This yields incomplete descriptions of the genes activated and deactivated, resulting on ill defined characterizations of the functions that account for the experiments. It is expectable that, where conventional methods for finding signatures from gene expression data are failing in capturing part of the functional information of the modules, methods based on gene sets [34], [35] and specifically those that consider the structure of the network [36], [37] will produce sounder results.

## Materials and Methods

### Signatures and co-expression modules

The L2L Microarray Database, accessible through a web portal [38], contains a collection of results derived from published microarray data. These results are essentially gene signatures and co-expression modules defined by the database curators or directly by the authors of the papers. Every microarray experiment in the collected publications generates lists of genes that are found to be characteristic of some condition or timepoint (see [22]). Typically, signatures are defined by the application of simple tests such as t-tests or other similar tests (see details in [22]). A total of 618 signatures defined as genes differentially expressed among a wide range of experimental conditions compared, from human microarray experimental results were downloaded from L2L database [22]. The signatures used here represent the following experimental conditions: 213 cancer, 405 non-cancer, 301 up-regulated, 243 down-regulated. A total of 507 modules of co-expression in cancer, defined as sets of genes co-expressing for a particular set of microarrays, were downloaded from L2L [22]. Co-expression modules have been also downloaded from L2L database [38], although the original ones can be found in the Module Networks site [39]. Co-expression modules were originally defined by bi-clustering methods (see details in [40]).

### Databases and interactome scaffold generation

The GO database was taken from Ensembl (release 54, May 2009). The KEGG database corresponds to the kegg50 release. Biocarta was downloaded by May 2009.

The program SNOW [24] (version 1.0) was used for the analyses. SNOW contains a database of PPI generated from the following public repositories: HRPD [41] (release 7 downloaded 31/03/2009), IntAct [42] (downloaded 31/03/2009), BIND [43] (release 2007-05-10), DIP [44] (release Hsapi20090126) and MINT [45] (release 2009/02/05). Entries in databases were mapped to Ensembl transcripts and genes. We used this collection of PPI data to generate two different types of interactomes (for both transcripts and genes): a non-filtered scaffold interactome, which include all the available PPIs, and a more confident, filtered scaffold interactome. The six top categories of experimental methods described in the Molecular Interaction Ontology [46] plus the categories *in vivo* and *in vitro* from HPRD were used as confidence measurements. Thus, only PPIs verified by at least two of these categories were considered in the filtered scaffold interactome.

Given a set of gene products, the sub-network defined by them can be easily determined by mapping all the members onto a scaffold interactome.

### Calculation of network parameters

**Figure 5:**
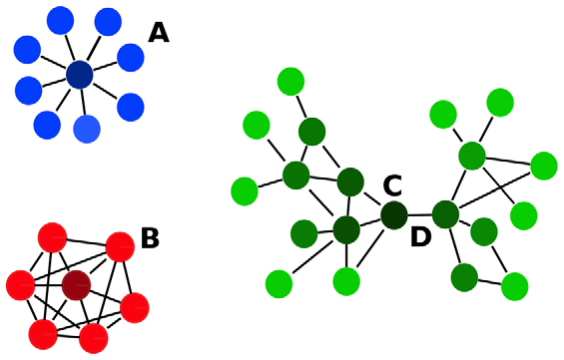
Local properties of the nodes represented by the different network parameters used. Connection degree was computed as the number of edges (interaction events) for a given node. **A** shows a node with a high connectivity. **B** has both high clustering coefficient and connectivity. **C** has a high betweenness. It is a hub because many shortest paths joining nodes pass through it. The connecting edge between two component (**D**) is known as the articulation point.

Different network parameters represent local and global network properties. Figure 5 schematizes the local properties of the nodes portrayed by the different parameters used. The properties used in this study are: Connection degree, Clustering coefficient, Betweenness centrality and components.

#### Connection degree

This parameter accounts for the number of partners of direct interaction a particular node has. For a given p, the connection degree is computed as the number of edges (interaction events). Figure 5A shows a node with value of connectivity of 8.0.

#### Clustering coefficient

This parameter not only accounts for the connectivity of a given node but also for the connectivity of the neighbourhood to which this node is connected. The Clustering coefficient of a node (C(ν)) was obtained by the formula:
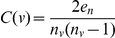
where e_n_ is the number of edges among the nodes connected to node ν, and n_ν_ is the number of neighbours of node ν. Figure 5A has the lowest possible clustering coefficient: 0, despite having high connectivity. On the other hand, Figure 5B has both high values of clustering coefficient, C(ν) = 0.6, and connectivity, C = 8.0.

#### Betweenness centrality

Is related to the concept of hub in a network and the capacity of traversing the network through many alternative paths connecting nodes situated in different extremes. A densely connected network does not necessarily imply many possibilities of traversing it. Betweenness centrality is related to the existence of hubs connecting different parts of the network. Betweenness centrality (C_B_ (ν)) of a node is obtained by applying the formula:
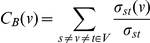
being σ_st_(ν), the number of shortest paths through a node and σ_st_, the total number of shortest paths in the graph. The shortest paths among nodes are calculated by Dijkstra algorithm [47], a widely used algorithm in network analysis. Relative betweenness centrality (rC_B_ (ν)) was calculated as:
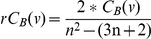
being n the total number of nodes in the graph. Node C in Figure 5 has a high betweenness given that many shortest paths joining nodes pass through it.

#### Components

A component in a graph is a group of nodes connected among them. Given a list of nodes connected among them, the minimum connection network (MCN) can be deduced by using the Dijkstra algorithm [47], which finds shortest the paths among all the nodes. The number of components can easily be added up once the MCN is derived.

The program SNOW [24], integrated now in the Babelomics package [48], is used for the calculation of all the network parameters above mentioned.

### PPI network enrichment analysis: Evaluating network parameters in the Minimal Connected Network

The network enrichment analysis consists on testing whether the parameters that describe a network are beyond their random expectations or not. When such parameters are significantly different from what it can be expected just by chance the network can then be considered to be a subset of the interactome enough connected to be considered a real network. The methodology has been previously published [24] and is briefly described below.

Given a list of nodes (proteins, genes or transcripts), the MCN joining them can easily be derived by mapping the nodes onto the scaffold interactome and finding the shortest paths among all the connected nodes. Thus, connection degree, betweenness centrality and clustering coefficient are parameters that can be measured for each node in the network. Consequently, a distribution for any of these parameters can be obtained for the MCN. Once these distributions are available, a simple Kolmogorov-Smirnov test can be used to check if one or several parameters of the network follow a distribution significantly different from the “random expectation”. The distributions for the “random expectations” of the network parameters of a MCN obtained for N nodes can be constructed by repeating (10,000 times in this case) the following steps: N proteins are randomly sampled from those contained in the reference interactome. They are mapped in the reference interactome and the corresponding MCN_RandList_ is obtained. The network parameters (connection degree, betweenness centrality and clustering coefficient) are used for constructing the corresponding distributions.

Then, the Kolmogorov-Smirnov test can be used to compare the parameter distributions of the MCN obtained from the problem list to their corresponding “random expectations”. The values of the studied parameters for a real sub-network should be significantly higher than the values obtained for the random (and consequently poorly connected) networks.

The number of components of the network can also be tested. This is a simpler case in which the distribution generated can directly be used to build a confidence interval. In this case, the real network should have significantly fewer components than the random network.

The program SNOW [24], now part of the Babelomics package [48], implements these calculations.

### Using external nodes

Exactly the same calculations can be performed for an extended MCN. This extension can be attained by using extra nodes, not included in the list of nodes to analyze, that connect two or more nodes in such list. The rationale for this is that often biological systems are poorly characterized and, consequently, lists of interest are not complete. For example, in cases of selection of proteins by expression profiling, non pre-selected proteins have been reported to be related to disease due to its inclusion into a network of PPIs [25], [26].The inclusion of such external nodes allows exploring the network space around the MCN and compensate possible nodes that remain undetected in a proteomics or microarray experiment or that remained unnoticed by annotators. This analysis is carried out as follows: for a list of N nodes mapped onto the interactome, all the E nodes that connect any two nodes in the list are found. Then, the MCN joining the N+E nodes is obtained and the corresponding network parameters are calculated. These values are compared by conducting a Kolmogorov-Smirnov test against the corresponding random expectations. The random expectation for a extended network of N nodes is found as follows: N nodes are sampled randomly from the interactome. Then, the Er nodes that connect any of these nodes are added to the random list. Then, the network parameters are calculated for the N+Er nodes. The procedure is repeated 10,000 times to obtain an empirical distribution of the parameters.

Again, the SNOW [24] program can also be used to calculate all the network parameters for the MCN and for the extended MCN.

## Supporting Information

File S1 **Signature parameters.** Excel file containing all the signatures analysed with the values of the four network parameters.
(XLS)

File S2 **Coexpression modules parameters.** Excel file containing all the coexpression modules analysed with the values of the four network parameters.
(XLS)
